# Signal transduction in mammalian oocytes during fertilization

**DOI:** 10.1007/s00441-015-2291-8

**Published:** 2015-10-09

**Authors:** Zoltan Machaty

**Affiliations:** Department of Animal Sciences, Purdue University, 915 W. State Street, West Lafayette, IN 47907 USA

**Keywords:** Oocyte, Signal transduction, Fertilization, Sperm, Embryo

## Abstract

Mammalian embryo development begins when the fertilizing sperm triggers a series of elevations in the oocyte’s intracellular free Ca^2+^ concentration. The elevations are the result of repeated release and re-uptake of Ca^2+^ stored in the smooth endoplasmic reticulum. Ca^2+^ release is primarily mediated by the phosphoinositide signaling system of the oocyte. The system is stimulated when the sperm causes the hydrolysis of phosphatidylinositol 4,5-bisphosphate (PIP_2_) into inositol 1,4,5-trisphosphate (IP_3_) and diacylglycerol (DAG); IP_3_ then binds its receptor on the surface of the endoplasmic reticulum that induces Ca^2+^ release. The manner in which the sperm generates IP_3_, the Ca^2+^ mobilizing second messenger, has been the subject of extensive research for a long time. The sperm factor hypothesis has eventually gained general acceptance, according to which it is a molecule from the sperm that diffuses into the ooplasm and stimulates the phosphoinositide cascade. Much evidence now indicates that the sperm-derived factor is phospholipase C-zeta (PLCζ) that cleaves PIP_2_ and generates IP_3_, eventually leading to oocyte activation. A recent addition to the candidate sperm factor list is the post-acrosomal sheath WW domain-binding protein (PAWP), whose role at fertilization is currently under debate. Ca^2+^ influx across the plasma membrane is also important as, in the absence of extracellular Ca^2+^, the oscillations run down prematurely. In pig oocytes, the influx that sustains the oscillations seems to be regulated by the filling status of the stores, whereas in the mouse other mechanisms might be involved. This work summarizes the current understanding of Ca^2+^ signaling in mammalian oocytes.

## Introduction

Prior to fertilization, mammalian oocytes are arrested at the metaphase stage of the second meiotic cell division. The meiotic block is characteristic of the entire animal kingdom and, although it may happen at various stages of the cell cycle, its role is to prevent entry into the embryonic cell cycles without the sperm (Dupré et al. 2011). The ovulated oocyte is a highly differentiated cell that, without fertilization, would die within 24–48 h. The fertilizing sperm, however, provides a stimulus that alleviates the meiotic arrest and activates the oocyte’s developmental program. During activation, the content of cortical granules is released into the perivitelline space. This triggers changes in the oocyte’s extracellular matrix, the zona pellucida, to prevent penetration by additional spermatozoa (Jaffe and Gould 1985). The activity of cell cycle regulatory proteins that maintain the arrest, such as cyclin-dependent kinase 1 (Cdk1; a component of the M-phase Promoting Factor) and Mitogen-Activated Protein Kinase (MAPK; part of the Cytostatic Factor) decreases, while that of others, such as the Anaphase Promoting Complex (APC) increases (Whitaker 1996; Nixon et al. 2002). As a result, the cell cycle resumes, meiosis is completed and after formation of the male and female pronuclei, the activated oocyte (now a 1-cell embryo) enters the first mitotic division. Activation is a remarkable process. It allows a differentiated cell to become totipotent and give rise to all the different cell types of a new organism. The transition is triggered by a highly intricate signal transduction mechanism that the sperm stimulates following sperm–oocyte fusion. This review describes the signaling pathway and discusses how it operates in mammalian oocytes to mediate the formation of an embryo, the founder of a new generation.

## The rise of calcium

It was Jacques Loeb who first suggested that oocyte activation involves changes in the concentration of ions in the ooplasm (Loeb 1899). His idea was based on the observation that sea urchin eggs started to develop parthenogenetically in the absence of sperm, simply by being bathed in seawater containing increased levels of ions. At a time when embryo development was explained with “vital forces”, not everybody was impressed; The *New York Times* referred to him simply as “a man of lively imagination”. The notion, however, was so fascinating that even Mark Twain wrote an essay about it titled “Dr. Loeb’s Incredible Discovery”. The calcium ion (Ca^2+^) was singled out by Lewis Victor Heilbrunn. Although the importance of Ca^2+^ in the contraction of skeletal muscle was demonstrated earlier (Ringer 1883), it was Heilbrunn who discovered that Ca^2+^ was the trigger not only for oocyte activation but also a great number of additional biological processes including ciliary movement, neurotransmitter release, increase or decrease in cell respiration and cell aging (Heilbrunn 1937). Considered by many in his time as a ‘calcium maniac’ (Shreeve 1983), Heilbrunn proposed that the breakdown of the nuclear membrane in the oocyte of the ragworm *Nereis* following fertilization or parthenogenetic activation was due to the release of Ca^2+^ inside the cell (Heilbrunn and Wilbur 1937). The increase in the free Ca^2+^ concentration during fertilization was quantitated in the eggs of another marine invertebrate, the sea urchin *Arbacia punctulata* (Mazia 1937). It was then demonstrated that treating sea urchin eggs with a Ca^2+^ ionophore that induced the release of Ca^2+^ from the intracellular stores caused parthenogenetic activation (Steinhardt and Epel 1974). The role of Ca^2+^ as the trigger of oocyte activation was proved when in medaka oocytes fertilization was shown accompanied by an elevation in the intracellular free Ca^2+^ concentration (Ridgway et al. 1977) and inhibition of this increase in sea urchin eggs blocked changes associated with activation (Zucker and Steinhardt 1978; Whitaker and Steinhardt 1982). Since these early studies, it has been firmly established that in virtually all animals it is Ca^2+^ that induces activation of the dormant oocyte. In most species, the sperm triggers a single elevation in the oocyte’s intracellular free Ca^2+^ concentration. The increase generally originates at the site of sperm entry and travels across the oocyte as a propagating Ca^2+^ wave (Gilkey et al. 1978). However, in mammals and some other species, including nemertean worms, ascidians, some annelids and arthropods, a series of low-frequency Ca^2+^ oscillations take place in the ooplasm at fertilization (Stricker 1999; Kashir et al. 2013a). In these cases, the first sperm-induced Ca^2+^ transient also arises near the site of sperm attachment and propagates as a wave across the entire oocyte. The initiation site of subsequent waves may undergo a shift: in mouse oocytes, it translocates from the point of sperm entry to the vegetal cortex (Deguchi et al. 2000).

Oscillatory Ca^2+^ signals have physiological advantages over static Ca^2+^ increases and they affect subsequent development. The repetitive behavior provides a means to deliver prolonged Ca^2+^ signals to targets without the deleterious effects of sustained Ca^2+^ elevations. The amplitude, frequency and duration of the sperm-induced Ca^2+^ signals encode crucial information and have a profound effect on peri-implantation development in addition to effects on the immediate events of oocyte activation (Ozil and Huneau 2001). Although a single increase in the intracellular free Ca^2+^ concentration can promote parthenogenetic development, freshly ovulated oocytes showed limited cell cycle progression and mRNA recruitment following activation with a single Ca^2+^ stimulus and only after aging could a single Ca^2+^ rise stimulate these critical events (Jones 1998; Ozil et al. 2005). By manipulating the number of Ca^2+^ transients in fertilized mouse oocytes, it was demonstrated that the first few Ca^2+^ transients were able to induce development to the blastocyst stage but fewer offspring were born from these embryos, indicating that the developmental competence of the blastocysts was reduced. Microarray analysis of global gene expression patterns in these embryos revealed that ∼20 % of the genes were misregulated, particularly those involved in RNA processing, polymerase II transcription, cell cycle and cell adhesion (Ozil et al. 2006).

## How does the sperm trigger the Ca^2+^ rise?

**Fig. 1:**
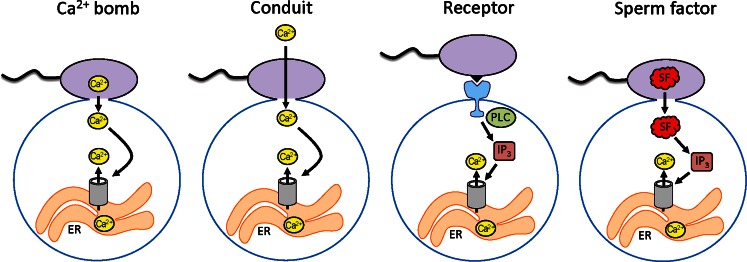
Schematic illustration of various hypotheses explaining the way the fertilizing sperm induces an elevation in the intracellular free Ca^2+^ concentration of the oocyte. The Ca^2+^ bomb hypothesis proposes that the sperm delivers a bolus of Ca^2+^ that causes Ca^2+^-induced Ca^2+^ release in the oocyte. According to the Ca^2+^ conduit model, the sperm facilitates Ca^2+^ entry from the extracellular medium. The receptor hypothesis suggests that Ca^2+^ release is induced when the sperm interacts with an oocyte receptor leading to the generation of IP_3_, the Ca^2+^ releasing second messenger. Finally, the sperm factor hypothesis claims that a factor from the sperm diffuses into the ooplasm and causes the production of IP_3_ to mobilize Ca^2+^

Once it was clarified that oocyte activation is stimulated by Ca^2+^, the next question that logically occurred was how the sperm triggers the Ca^2+^ elevation in the ooplasm? This issue, however, remained unresolved for a long time. Multiple hypotheses were proposed to explain the generation of the fertilization Ca^2+^ signal. The earliest model known as the “Ca^2+^ bomb hypothesis” postulated that the sperm introduces Ca^2+^ into the oocyte that sets off a wave of Ca^2+^-induced Ca^2+^ release (Fig. 1) (Jaffe 1983). However, the Ca^2+^ content of the sperm is not sufficient to trigger Ca^2+^ release and the hypothesis was subsequently modified to suggest that the sperm serves as a Ca^2+^ conduit, allowing Ca^2+^ from the extracellular medium to flow into the ooplasm (Jaffe 1991). The Ca^2+^ is then pumped into the endoplasmic reticulum, which results in the overloading of the stores and the release of luminal Ca^2+^. Even in this form, the theory did not stand the test of time. The injection of Ca^2+^ into the ooplasm fails to cause further Ca^2+^ release in many species, or to trigger repetitive Ca^2+^ oscillations in mammalian oocytes (Swann and Whitaker 1986; Swann and Ozil 1994). In addition, no local elevation in the cytoplasmic Ca^2+^ levels has been detected near the site of sperm–oocyte fusion (Jones et al. 1998); as it turned out, a Ca^2+^ entry takes place after (rather than before) the first Ca^2+^ transient (McGuinness et al. 1996).

According to the “receptor hypothesis”, the fertilizing sperm induces the Ca^2+^ oscillations by binding to a receptor on the surface of the oocyte plasma membrane. Just like hormone–receptor binding in somatic cells, the interaction between a sperm ligand and a receptor spanning the oolemma was proposed to activate a signaling pathway that ultimately leads to the release of Ca^2+^ from the endoplasmic reticulum. This hypothesis was supported by a number of observations and, for many years, it was the dominant model to explain generation of the fertilization Ca^2+^ signal (Jaffe 1990; Schultz and Kopf 1995). Although numerous publications suggest that oocytes contain a signaling pathway associated with cell surface receptors, there is no evidence that the sperm triggers oocyte activation via these pathways. The ligands and receptors identified so far on the surface of mammalian gametes are involved in the mediation of sperm–oocyte binding and fusion, with no role in stimulating Ca^2+^ release (Wassarman et al. 2005).

The “sperm factor hypothesis” proposes that oocyte activation is induced by a soluble factor in the sperm that is released into the oocyte at fertilization. In mouse, it was shown that fusion between the sperm and oocyte membranes precedes the first Ca^2+^ transient by 1–3 min, which is consistent with the notion that the sperm-resident factor needs time to move into the oocyte cytoplasm before it mobilizes Ca^2+^ from the internal stores (Lawrence et al. 1997). The finding that the injection of a crude extract isolated from the head of mammalian sperm is able to induce repetitive Ca^2+^ oscillations in mammalian oocytes also supports this hypothesis (Swann 1990; Wu et al. 1997; Machaty et al. 2000). It was also reported that sperm extracts from fish, frogs and chickens caused oscillations in mouse oocytes (Dong et al. 2000; Coward et al. 2003). In addition, oocytes can be activated by intracytoplasmic sperm injection, where membrane interaction between the sperm and oocyte is bypassed during the injection process. This also argues in favor of the proposal that it is a factor in the sperm head that initiates the Ca^2+^ changes at fertilization.

## The signaling pathway

The fertilizing sperm can generate the initial Ca^2+^ transient even in the absence of extracellular Ca^2+^; this led to the realization that the origin of Ca^2+^ that activates the oocyte is intracellular (Gilkey et al. 1978). In many cells, the intracellular stores reside in the smooth endoplasmic reticulum. Ca^2+^ is loaded into the lumen of the stores by sarcoplasmic/endoplasmic reticulum Ca^2+^ ATPases (SERCA pumps) and stored while attached to Ca^2+^-binding proteins (Berridge 2002). During signaling, the stored Ca^2+^ is released into the cytosol through Ca^2+^ release channels. Two types of channels, which also function as receptors for their respective Ca^2+^ mobilizing ligands, are available for Ca^2+^ release. The inositol 1,4,5-trisphosphate (IP_3_) receptor is a protein complex of four subunits that surround the channel pore (Mikoshiba 1993). Each subunit can bind one IP_3_ molecule and binding leads to the release of Ca^2+^ from the store. The sensitivity of the IP_3_ receptor to IP_3_ is biphasic: it is the greatest in the physiological range between 0.5 and 1 μM (Hajnóczky and Thomas 1994). The receptor is also gated by Ca^2+^. Its cytoplasmic region has at least one binding site for Ca^2+^ (Mignery and Südhof 1990) and experimental data indicate that the receptor shows biphasic sensitivity to cytoplasmic Ca^2+^. Thus, regulation of the receptor is complex: it is opened by IP_3_ but is also desensitized by it, while low and high Ca^2+^ concentrations make it relatively insensitive to otherwise activating IP_3_ levels. The other type of Ca^2+^ release channels/receptors is the ryanodine receptor. It is also a homotetramer of four subunits and its opening is controlled by cyclic adenosine diphosphate ribose (cADPR), by Ca^2+^ itself and in skeletal muscle by electromechanical coupling to the dihydropiridine receptor located in the plasma membrane (Coronado et al. 1994).

In mammalian oocytes, the generation of the fertilization Ca^2+^ transients is mediated by the phosphoinositide signaling system. Such a system produces a signal when IP_3_ binds its receptor, leading to the opening of the channel and the release of Ca^2+^ into the cytosol. IP_3_ is produced when phospholipase C (PLC), a cytoplasmic enzyme, cleaves phosphatidylinositol 4,5-bisphosphate (PIP_2_), a phosphoinositide, into IP_3_ and diacylglycerol (DAG) (Miyazaki et al. 1993). Currently, there are 13 known mammalian phosphoinositide-specific PLC isozymes; their classification is based on structure and regulation. They include 4 types of PLCβ (PLCbeta), 2 types of PLCγ (PLCgamma), 3 types of PLCδ (PLCdelta), PLCε (PLCepsilon), PLCζ (PLCzeta) and 2 types of PLCη (PLCeta) (Bunney and Katan 2011). All isozymes exhibit characteristic X and Y catalytic domains that together form the active site responsible for cleaving PIP_2_. They also contain various combinations of regulatory domains that target the enzymes to their respective activators or substrates; these include pleckstrin homology (PH) domains, Src homology 2 (SH2) domains and constant or conserved region 2 (C2) domains. The mechanism of activation varies depending on the specific combination of regulatory domains present in each PLC isozyme. PH domains bind phosphoinositides such as PIP_2_ and PIP_3_ and thus they typically serve to target PLC to the plasma membrane where most phosphoinositides are located. In addition, they can also mediate interaction with heterotrimeric G proteins (Camps et al. 1992). SH2 domains interact with receptor tyrosine kinases and also with non-receptor tyrosine kinases such as Src (Weiss 1993). Finally, C2 domains bind Ca^2+^ and bestow phospholipid-binding properties to the enzyme.

Several lines of evidence indicate the involvement of the phosphoinositide signaling cascade during fertilization. Biochemical analyses in sea urchin and frogs have shown an increased turnover of polyphosphoinositides and an elevation in IP_3_ levels after gamete interaction (Turner et al. 1984; Snow et al. 1996). In addition, a monoclonal antibody against the IP_3_ receptor inhibits the sperm-induced Ca^2+^ transients (Miyazaki et al. 1992), while sustained microinjection of IP_3_, or adenophostin (an IP_3_ analogue) can also trigger Ca^2+^ oscillations in mammalian oocytes (Swann 1994; Jones and Nixon 2000). Further evidence also shows that the PLC inhibitor U73122 blocks activation in the sea urchin and mouse (Dupont et al. 1996; Lee et al. 1998). Finally, in mouse and bovine oocytes, there is a significant down-regulation of the IP_3_ receptors, i.e., their number decreases markedly at the time of fertilization (Brind et al. 2000; Jellerette et al. 2000). Normally, this occurs only after a substantial rise in the IP_3_ concentration, implying that at fertilization the sperm stimulates an increase in IP_3_ levels in the oocyte cytoplasm. Initial investigations were focused on oocyte-resident PLCs and the presence of PLCβ, PLCγ and PLCδ was demonstrated in the female gamete. PLCβ isoforms are generally coupled to membrane receptors via a G protein, whereas γ isoforms are directly linked to receptor tyrosine kinases. Microinjection of a non-hydrolyzable analog of GTP that stimulates G proteins (GTPγS) caused activation in sea urchin eggs (Turner et al. 1986) and induced regenerative Ca^2+^ rises in some mammalian oocytes (Miyazaki 1988; Swann 1992; Fissore et al. 1995). Also, overexpression of the G protein-coupled muscarinic receptor in frog, mouse and pig oocytes led to activation after exposure of the oocytes to acetylcholine, the receptor’s ligand (Kline et al. 1988; Williams et al. 1992; Machaty et al. 1997). These findings suggested that the pathway that mediated Ca^2+^ release might include a PLCβ connected to a G protein-coupled receptor.

An alternative signaling mechanism that was implicated by experimental data involved PLCγ and an associated receptor tyrosine kinase. Overexpression and subsequent stimulation of such receptors in frog and mouse oocytes leads to activation (Yim et al. 1994; Mehlmann et al. 1998), which seems to support the theory. However, recombinant SH2 domains of PLCγ block PLCγ activation by the receptor but they cannot inhibit Ca^2+^ release at fertilization (Mehlmann et al. 1998; Runft et al. 1999), which argues against the involvement of PLCγ in the signaling process at fertilization. In addition, when the phosphoinositide signaling system is artificially activated using GTPγS, or non-hydrolyzable analogs of IP_3_, the Ca^2+^ signal that is generated is still a far cry from the low-frequency Ca^2+^ oscillations associated with mammalian fertilization (Miyazaki et al. 1990; Swann and Ozil 1994; Galione et al. 1994; Machaty et al. 1997). Taken together, these data indicate that the oocytes contain a phosphoinositide signaling pathway; however, the exact mechanism that mediates its activation at fertilization has not been identified by these studies.

## Finding PLCζ

As described above, a number of observations supported the idea that the sperm might stimulate the phosphoinositide signaling pathway by introducing a soluble factor into the oocyte after fusion. This led to a quest to identify the oocyte activating factor but the efforts proved futile for a long time. The molecule was shown to be a protein since heat treatment or proteases abolished its Ca^2+^-inducing activity; it was also believed to have a high molecular mass and be present in cytosolic extracts (Swann 1990). Mammalian sperm extracts showed high PLC enzyme activity in biochemical assays, which suggested that the sperm factor that activates the oocyte might itself be a PLC (Rice et al. 2000). Spermatozoa of mammalian species express several PLC isoforms including PLCβ, -γ and -δ (Fukami 2002); however, when the recombinant forms of these proteins were injected into oocytes, they were unable to induce Ca^2+^ oscillations at physiological levels (Mehlmann et al. 2001). And because chromatographic fractionation of sperm extracts indicated that none of the known PLC isoforms were present in the fraction that were able to induce regenerative Ca^2+^ rises (Parrington et al. 2002), the idea came that the sperm factor might be a novel PLC.

The analysis of mouse expressed sequence tag (EST) databases led to the identification and eventual amplification of a new, testis-specific PLC variety, termed PLCζ (Saunders et al. 2002). With its ∼74 kDa molecular weight, it is the smallest known mammalian PLC. Recombinant PLCζ, or its complementary RNA (cRNA), are both able to induce regenerative Ca^2+^ oscillations in mouse oocytes similar to those found at fertilization (Saunders et al. 2002; Cox et al. 2002; Kouchi et al. 2004). Furthermore, when injected into human and pig oocytes, PLCζ cRNA can stimulate embryo development to the blastocyst stage (Rogers et al. 2004; Yoneda et al. 2006). Immunodepletion with an anti-PLCζ antibody suppressed the extracts’ ability to induce Ca^2+^ release in mouse oocytes or sea urchin egg homogenates (Saunders et al. 2002). The presence of PLCζ orthologues has been demonstrated in the sperm of other mammalian species, including hamster, pig, horse, monkey and human (reviewed by Nomikos et al. 2013). In mice, the protein is localized in the postacrosomal region of the perinuclear theca, a condensed layer of cytosolic proteins that covers the nucleus (Young et al. 2009); in cattle, it resides in the equatorial region of the sperm head (Yoon and Fissore 2007). This is the localization that is expected from a sperm-resident factor that needs to gain rapid access to the ooplasm after gamete fusion to mobilize Ca^2+^ (Lawrence et al. 1997). The use of tagged versions of the protein has indicated that approximately 40 fg PLCζ is able to trigger repetitive Ca^2+^ transients in mouse oocytes and this is the amount estimated to be present in a single sperm (Saunders et al. 2002). Injection of PLCζ into mouse oocytes causes a down-regulation of IP_3_ receptors similar to that seen at fertilization, indicating that PLCζ generates IP_3_ in the cytoplasm (Lee et al. 2010). When spermatozoa from transgenic mice showing reduced PLCζ expression were used to fertilize oocytes, the Ca^2+^ oscillations generated in the ooplasm stopped prematurely (Knott et al. 2005). Although these mice were not completely infertile, they produced markedly reduced litter sizes following mating. Sperm of human patients that failed to activate the oocyte also had deficiencies in their PLCζ: they either showed reduced or complete absence of the enzyme, or possessed deleterious mutations within the catalytic X and Y domains (Yoon et al. 2008; Kashir et al. 2012). The analysis of sperm that is completely devoid of PLCζ would provide invaluable information regarding the role of the protein in Ca^2+^ signaling at fertilization. Unfortunately, although PLCζ-knockout mice have already been created, they are unable to produce sperm; the germ cells in the testes of such animals develop only up to the round spermatid stage (Ito et al. 2010). It was demonstrated some time ago (Kimura and Yanagimachi 1995) that microinjection of round mouse spermatids is unable to activate oocytes (a potential explanation to this might be that, according to one study, PLCζ expression in mice begins in elongated spermatids only [Yoneda et al. 2006]); hence, these cells cannot be used to determine the importance of PLCζ in fertilization. Nevertheless, despite the absence of this ultimate test, the data listed above strongly argue in favor of the idea that, in mammals, PLCζ has a central role in the generation of the Ca^2+^ signal to activate the oocyte and stimulate embryo development.

## PLCζ characteristics

The PLCζ orthologues identified in various mammalian species are all similar in size (Swann et al. 2006). Surprisingly, they lack the N-terminal PH domain that is present in other PLC isoforms and instead contain two pairs of EF hand domains, followed by the XY catalytic domain and a C2 domain at the C-terminus. PLCζ is much more potent than other PLC isoforms in generating Ca^2+^ oscillations; its closest homologue, PLCδ1 triggers oscillations only when it is present in mouse oocytes at concentrations higher than 1 pg (Saunders et al. 2002; Nomikos et al. 2011). As in other isoforms, the XY catalytic domain is responsible for enzymatic activity; a point mutation in this domain causes a loss in the enzyme’s ability to generate IP_3_ and induce Ca^2+^ oscillations (Nomikos et al. 2011). The activity is not species specific as cRNA of various mammalian or non-mammalian PLCζ orthologues can cause oscillations in mouse oocytes (Cox et al. 2002; Coward et al. 2005).

The EF hands possess Ca^2+^ binding residues and provide the enzyme with high Ca^2+^ sensitivity; deletion or mutation of conserved Ca^2+^ binding residues in this region abolish Ca^2+^-induced PLC activity. PLCζ is 100-fold more sensitive to Ca^2+^ than PLCδ and this is believed to be a major reason why the enzyme is highly effective in oocytes. Even at resting Ca^2+^ levels, PLCζ shows half maximal activity and, with rising cytosolic Ca^2+^ concentrations, its enzymatic activity increases markedly (Nomikos et al. 2005). Thus, following gamete fusion, when PLCζ diffuses into the ooplasm, a small increase in cytosolic Ca^2+^ causes a significant elevation in PLC activity, leading to the generation of large amounts of extra IP_3_. This creates a positive feedback loop of IP_3_ production and Ca^2+^ release in fertilized oocytes, setting the stage for the regenerative Ca^2+^ signal.

C2 domains generally bind Ca^2+^ (Nalefski and Falke 1996) and Ca^2+^ binding to the C2 domain is typically crucial for enzyme activity (Zheng et al. 2000). However, the C2 domain of PLCζ has no predicted Ca^2+^ binding site. Deletion of this domain does not alter enzyme activity of PLCζ, nevertheless it abolishes its ability to induce Ca^2+^ oscillations, indicating that the C2 domain is critical for PLCζ function (Nomikos et al. 2005). Another segment, the XY-linker that joins together the X and Y catalytic domains, also has a major impact on PLCζ function. As mentioned before, unlike other isoforms, PLCζ does not have a PH domain that typically functions to bind PIP_2_ at the plasma membrane. It was proposed, however, that positively charged residues within this region might target the enzyme to PIP_2_, possibly via electrostatic interactions (Nomikos et al. 2007). A decrease in the net positive charge of the X-Y linker, or the deletion of the entire linker, caused a decline in the enzyme’s ability to bind PIP_2_ in vitro or to induce Ca^2+^ oscillations after microinjection into oocytes (Nomikos et al. 2011). There are also distinct variations in the size of the X–Y linker between species. It is the shortest in humans and the longest in the *Cynomolgus* monkey (Swann et al. 2006); these differences may explain the diverse potency of PLCζ orthologs to generate Ca^2+^ transients (Saunders et al. 2007). Finally, the X–Y linker also possesses a predicted nuclear localization signal sequence that may be important in the control of PLCζ function (Larman et al. 2004).

## PLCζ localization in the sperm

According to the sperm factor hypothesis, a compound diffuses from the sperm into the ooplasm and causes Ca^2+^ release from the endoplasmic reticulum. The Ca^2+^ oscillations begin soon after the fusion of the gametes; in the mouse, the elapsed time is approximately 1–3 min (Lawrence et al. 1997; Jones et al. 1998); in the hamster, it is shorter, about 10 s (Miyazaki 1991). During this time (the so-called latent period), the sperm factor is supposed to get into the ooplasm and initiate the mobilization of Ca^2+^. This means that the factor must reside in the sperm at a location that provides easy access to the ooplasm. It is believed that the most ideal localization for the oocyte-activating factor is the post-acrosomal region of the perinuclear theca, a condensed layer of cytosolic proteins surrounding the nucleus of the sperm (Yanagimachi 1994). Immunofluorescent analysis of mouse spermatozoa determined that PLCζ is localized in the post-acrosomal region of the sperm head (Fujimoto et al. 2004); this region also seems to possess the ability to activate oocytes after intracytoplasmic sperm injection (Kimura et al. 1998; Perry et al. 2000). Importantly, this is the area that is exposed to the oocyte cytoplasm following fusion of the sperm’s equatorial region with the oolemma. In other species, it was found in the equatorial or acrosomal region (Yoon and Fissore 2007; Kashir et al. 2013b), while in equine sperm, PLCζ also resided in the principal piece of the tail (Bedford-Guaus et al. 2011). This latter finding was quite unexpected; however, because microinjection of the equine sperm tail caused Ca^2+^ oscillations into mouse oocytes (Bedford-Guaus et al. 2011), this further strengthened the idea that PLCζ is the molecule that triggers activation.

Solubility of PLCζ may be another aspect that influences its function as an oocyte-activating factor. Early studies in the hamster and swine determined that cytosolic extracts of the spermatozoa contained the active factor (Swann 1990); later experiments in the mouse, however, asserted that the sperm heads retained the activity after the removal of the soluble cytosolic fraction (Kimura et al. 1998; Perry et al. 2000). This seems to indicate differences among species and also variations in the solubility of PLCζ (solubility here refers to the ability to move via diffusion in the oocyte cytosol, it does not mean that it can be extracted into an aqueous solution). In hamster, the initial Ca^2+^ transient begins within seconds following gamete fusion (Miyazaki 1991) and much of PLCζ appears to exist in a soluble form in hamster sperm (Swann 1990) facilitating easy access to PIP_2_ and rapid Ca^2+^ mobilization in the oocyte cytoplasm. In mice, on the other hand, the Ca^2+^ oscillations are initiated several minutes after sperm-oocyte fusion and this relatively long latent period may be the consequence of the low solubility of mouse PLCζ that requires a longer time to move into the ooplasm from the sperm head. This idea is supported by the observation that, during isolation, the mouse sperm cytosol does not retain the oocyte-activating factor and more elaborate approaches are necessary for its extraction (Perry et al. 2000). Porcine PLCζ has been found in both soluble and insoluble fractions (Kurokawa et al. 2005). Based on these observations, an idea has been formulated, claiming that soluble PLCζ is located in the equatorial region of the sperm head and, due to its easy access to the ooplasm, it stimulates Ca^2+^ oscillations rapidly following gamete fusion. In contrast, insoluble PLCζ localizes in the postacrosomal region and mobilizes Ca^2+^ in a somewhat belated manner, once incorporation of the sperm head in the oocyte cytoplasm is at a more advanced stage (Kashir et al. 2014).

## PLCζ action in the oocyte

**Fig. 2:**
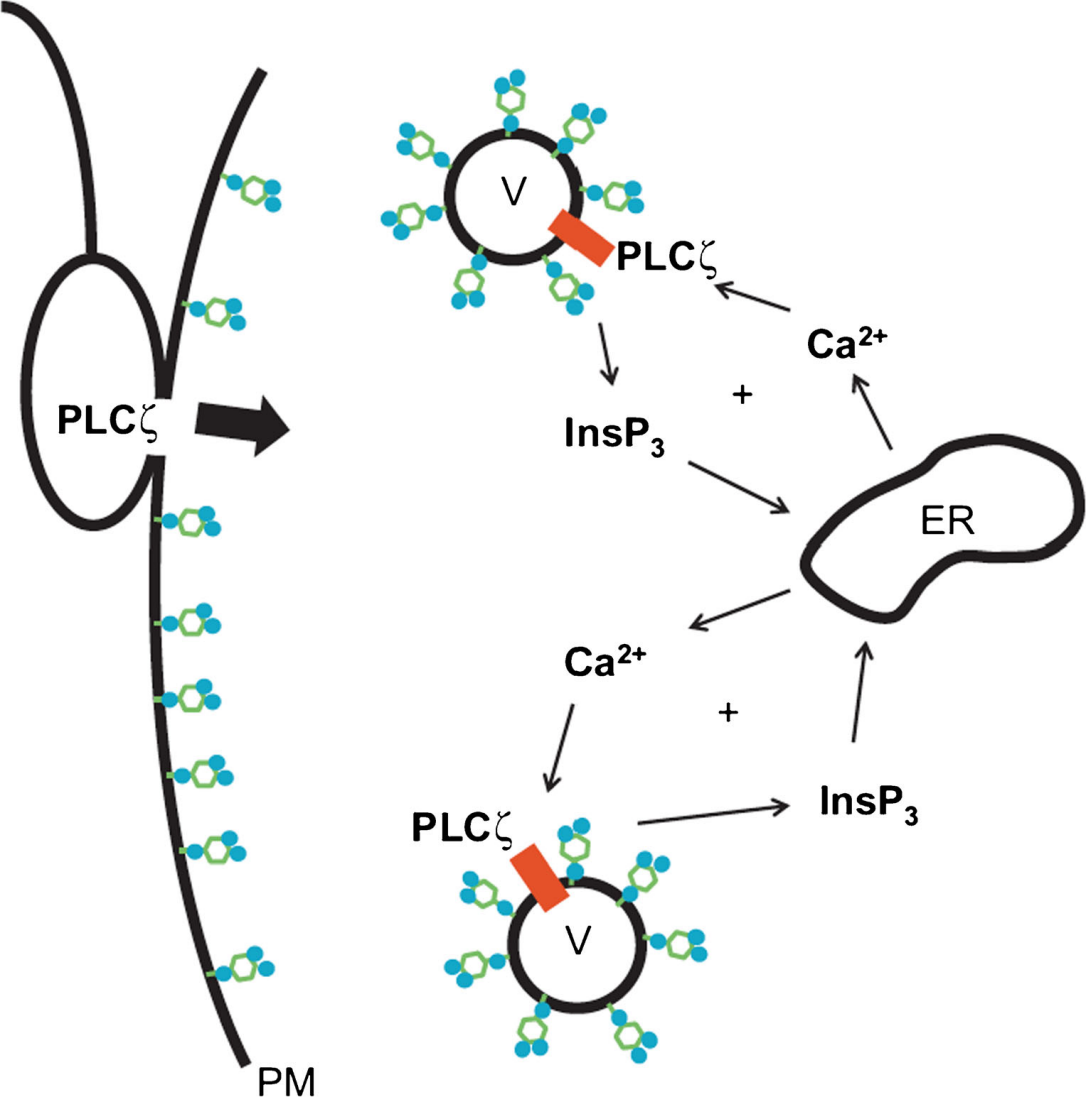
Hypothetical mechanism of PLCζ-induced Ca^2+^ release. Following gamete fusion, PLCζ diffuses into the ooplasm and hydrolyses PIP_2_ located in the membrane of cytoplasmic vesicles. It is possible that it binds to a yet-to-be-identified protein in the vesicular membrane (symbolized by a *red rectangle*). The IP_3_ generated as a result of the hydrolysis moves to the endoplasmic reticulum and induces the release of stored Ca^2+^. Elevated Ca^2+^ levels lead to increased PLCζ activity, which, through a positive feedback loop, stimulates further increase of Ca^2+^ and IP_3_ (from Swann and Lai 2013, with permission)

PLC enzymes generate IP_3_ by cleaving the phospholipid PIP_2_. Because PIP_2_ resides solely in biological membranes, one would expect PLCζ to accumulate in the plasma membrane where most of the cells’ PIP_2_ is located. However, fluorescently tagged mouse PLCζ localized in the cytoplasm instead of below the oolemma (Yu et al. 2012). In addition, there is no decrease in the PIP_2_ concentration at the plasma membrane in mouse oocytes at fertilization (Halet et al. 2002) and the level of DAG, the other product of PIP_2_ hydrolysis, does not increase at the plasma membrane during fertilization or after PLCζ injection (Yu et al. 2008). This apparent contradiction is solved in light of the findings that PIP_2_ in mouse oocytes resides not only in the plasma membrane but also in the membrane of vesicles inside the oocyte cortex (Yu et al. 2012). By means of immunocytochemistry, it was determined that PLCζ localized in the same vesicular structures and after PLCζ injection these vesicles displayed decreased PIP_2_ levels. Targeting an inositol phosphate phosphatase to the plasma membrane also supported these observations. The expression of such a phosphatase in mouse oocytes reduced the amount of plasma membrane-resident PIP_2_ and entirely abolished the Ca^2+^ transients triggered by the microinjection of PLCδ1 without affecting the sperm- or PLCζ-induced Ca^2+^ oscillations. This also explains previous reports that extracts made of boar sperm were able to generate IP_3_ most effectively in the subcellular fraction of sea urchin egg homogenates that were rich in yolk vesicles (Rice et al. 2000). This suggests that PLCζ uses a unique signaling cascade to mobilize Ca^2+^ during fertilization when it hydrolyzes PIP_2_ in intracellular membranes. The potential mechanism that PLCζ uses to induce Ca^2+^ release in oocytes is shown in Fig. 2.

In mammalian oocytes, the sperm-induced Ca^2+^ signal oscillates for an extended period of time. In the mouse, the oscillations cease after about 3–4 h, which coincides with the formation of the male and female pronuclei. The termination of the oscillations was proposed to be due to the sequestration of PLCζ into the forming pronuclei (Marangos et al. 2003) and a number of observations support this notion. It has been shown that, if pronuclear formation is inhibited, the oscillations continue indefinitely. In addition, the oscillations are absent while the pronuclei exist but at the onset of mitosis they resume as the nuclear envelopes break down. Transferring of the male or female pronucleus from fertilized oocytes causes Ca^2+^ oscillations in the cytoplasm of unfertilized oocytes while pronuclei of parthenogenetically activated oocytes are unable to do so (Kono et al. 1996). Furthermore, immunocytochemical experiments indicate that recombinant mouse PLCζ accumulates in the pronuclei upon the cessation of the oscillations (Larman et al. 2004; Yoda et al. 2004). Positively charged amino acid residues within the X–Y linker region are probably responsible for nuclear localization as mutation of these residues to negatively charged ones results in a loss of the nuclear translocation ability and in the persistence of oscillations after pronuclear formation (Larman et al. 2004). This suggests that nuclear sequestration of PLCζ is the reason for the cessation of the oscillations and it also explains why the oscillations resume at nuclear envelope breakdown, when the first mitotic division begins. Interestingly, nuclear sequestration of PLCζ seems to be characteristic in the mouse only, as bovine, rat and human PLCζ do not accumulate in the pronuclei, even in mouse oocytes following ectopic expression (Ito et al. 2008). Furthermore, rat PLCζ does not accumulate in the pronuclei of rat zygotes but mouse PLCζ does. It has also been reported that, in bovine and rabbit zygotes, the oscillations continue beyond pronucleus formation (Fissore et al. 1992; Fissore and Robl 1993). This indicates that additional factors may also control the Ca^2+^ signal and currently it is unclear how the Ca^2+^ oscillations in species other than the mouse are terminated.

## Other proposed sperm factors

A number of additional molecules have also been proposed to serve as a sperm-resident activating factor. The first candidate was “oscillin”, a protein isolated by serial chromatographic purification from hamster sperm. The protein seemed to be an oscillogen, as it co-migrated with the ability of the extract to trigger Ca^2+^ oscillations in oocytes (Parrington et al. 1996). However, recombinant oscillin did not cause Ca^2+^ oscillations in oocytes, indicating that it was not the active factor in the sperm (Wolosker et al. 1998). Another candidate sperm factor was tr-kit, a truncated form of the c-kit receptor (Sette et al. 1997). In mouse oocytes, tr-kit induced parthenogenetic activation and it was suggested that it stimulated PLCγ1 through phosphorylation by Fyn, a Src-like kinase (Sette et al. 2002). Nevertheless, although its action is inhibited by a PLCγ SH3 construct, the same construct has no effect on sperm-induced oocyte activation (Mehlmann et al. 1998). In addition, there is no evidence to indicate that tr-kit is able to induce regenerative Ca^2+^ oscillations, which would be an expectation from a bona fide sperm factor.

The latest addition to the list of potential sperm factors is the postacrosomal sheath WW domain-binding protein (PAWP). WW domains are small functional modules found in many signaling and structural proteins and are known to mediate protein–protein interactions. They are named after their two signature tryptophan (W) residues that play an important role in the domains’ function. Interaction between WW domain-containing proteins and their ligands is important for a great number of cellular events such as transcriptional activation, cell cycle control and ubiquitin ligation (Sudol and Hunter 2000; Macias et al. 2002). WW domains fall into two major groups (Group I and Group II) based on their ligand preferences, PAWP specifically binds to Group I WW domains (Wu et al. 2007a). PAWP expression begins during spermatid elongation in humans, rhesus monkey, mice, cows, pigs and rabbits (Wu et al. 2007a, b; Aarabi et al. 2014a) and the expressed protein resides in the postacrosomal sheath of the perinuclear theca, a localization that allows rapid access to the ooplasm. It has also been demonstrated that, after sperm–oocyte fusion, PAWP is released into the oocyte cytoplasm (Wu et al. 2007a, b). Microinjection of recombinant PAWP into *Xenopus* oocytes causes Ca^2+^ release (Aarabi et al. 2010), while in *Xenopus*, porcine, bovine and macaque oocytes, it was shown to trigger cell cycle progression and pronuclear formation (Wu et al. 2007a). In addition, the recombinant protein, or its cRNA, has been shown to trigger Ca^2+^ oscillations in mouse and human oocytes. The oscillations are blocked by co-injection of a peptide derived from the WWI domain-binding motif of PAWP that acts as a competitive inhibitor (Aarabi et al. 2014a). This inhibitory peptide is also able to block the Ca^2+^ transients after intracytoplasmic sperm injection, which implies that PAWP is involved in oocyte activation at fertilization. Correlative analyses in humans and livestock species have shown that inadequate amounts of PAWP in spermatozoa are associated with low fertility, possibly because such sperm are unable to trigger oocyte activation (Aarabi et al. 2014b). It has been suggested that, once PAWP is released into the oocyte, it interacts with Group I WW domain-containing proteins, such as the yes-associated protein (YAP). These proteins are highly expressed in oocytes and possess a SH3 binding motif (Chen and Sudol 1995); they may in turn activate PLCγ noncanonically, via its SH3 domain (Aarabi et al. 2014b). Such activation of PLCγ, i.e., via its SH3 instead of SH2 domain, has been observed in *Xenopus* oocytes and human neurons (Browaeys-Poly et al. 2007; Reynolds et al. 2008). However, since the microinjection of fusion proteins containing the SH3 domain of PLCγ did not inhibit fertilization in mouse oocytes (Mehlmann et al. 1998), it is still not clear what signaling cascade PAWP might use to trigger Ca^2+^ oscillations. In addition, the ability of mouse PAWP to trigger repetitive Ca^2+^ transients could not be verified by others (Nomikos et al. 2014, 2015b). Furthermore, in a most recent study, human PAWP was not able to induce Ca^2+^ oscillations in mouse oocytes and the PAWP-derived inhibitory peptide was also unable to block sperm-induced Ca^2+^ oscillations (Nomikos et al. 2015a). Detailed analyses of PAWP structure and function are also missing (Kashir et al. 2015); because of these reasons, the recognition of PAWP as the sperm-derived molecule that causes oocyte activation at fertilization requires further verification.

## Ca^2+^ influx

Once the Ca^2+^ signal is initiated, in mammalian oocytes it takes the form of repetitive Ca^2+^ oscillations. It is not completely clear what causes the signal to oscillate; the complex regulation and basic feedback properties of the IP_3_ receptor are usually listed as the most likely causes (Berridge and Galione 1988). In addition, the oscillations also depend on Ca^2+^ influx. After each Ca^2+^ rise, the resting Ca^2+^ level is rapidly restored. SERCA pumps move Ca^2+^ back into the endoplasmic reticulum (Kline and Kline 1992) and Ca^2+^ uptake by mitochondria may also be significant (Eisen and Reynolds 1985). At the same time, plasma membrane Ca^2+^ ATPase (PMCA) pumps and Na^+^/Ca^2+^ exchangers in the plasma membrane are also available for Ca^2+^ removal (Carroll 2000). In fact, a substantial efflux of Ca^2+^ was demonstrated in sea urchin eggs, frog and mouse oocytes after Ca^2+^ release (Steinhardt and Epel 1974; Shapira et al. 1996; Pepperell et al. 1999) and this outward Ca^2+^ current might be so substantial that the entry of extracellular Ca^2+^ becomes necessary to compensate for the loss. The need for Ca^2+^ entry to sustain the oscillations was first demonstrated in hamster oocytes. In these cells, the repetitive hyperpolarizations in the membrane potential (that were caused by a K^+^ conductance activated by the sperm-induced Ca^2+^ transients) were reduced in frequency and ultimately stopped upon superfusion with Ca^2+^-free medium (Igusa and Miyazaki 1983). In a similar manner, the train of Ca^2+^ spikes in fertilized mouse oocytes slows down or stops if Ca^2+^ is removed from the extracellular medium (Kline and Kline 1992; Shiina et al. 1993). Additional data supporting the function of a Ca^2+^ influx mechanism during fertilization were provided by experiments using dithiothreitol (DTT), a sulfhydryl reducing agent. In unfertilized mouse oocytes, DTT is able to stimulate the influx of divalent cations (including Ca^2+^), whereas in fertilized ones it increases the frequency of the Ca^2+^ oscillations (Cheek et al. 1993). This also implies that Ca^2+^ entry has an important role in signaling at fertilization. The link between the Ca^2+^ influx and the Ca^2+^ transients has been analyzed in fertilized mouse oocytes. According to the study, the rising phase of each transient is followed by an increase in Ca^2+^ entry, while the influx weakens (but still persists) between the transients (McGuinness et al. 1996). Results from another report also indicated that, during the initial Ca^2+^ release in sperm extract-injected mouse oocytes, a Ca^2+^ influx is generated and persists throughout the oscillations (Mohri et al. 2001). These observations all support the concept that the sperm-induced Ca^2+^ oscillations are associated with a Ca^2+^ influx across the plasma membrane and that the extracellular Ca^2+^ is essential for the refilling of the Ca^2+^ stores (Miyazaki 1991). They also seem to suggest that the filling status of the stores controls the influx: the release of Ca^2+^ from the endoplasmic reticulum apparently triggers the Ca^2+^ entry mechanism. Other potential mechanisms to mediate the Ca^2+^ influx initially included voltage-operated channels but they were quickly ruled out. Hamster oocytes contain voltage-gated Ca^2+^ channels (Miyazaki and Igusa 1981); however, as sustained hyperpolarization of the oolemma increases the frequency of the sperm-induced hyperpolarization responses, their involvement is highly questionable (Miyazaki 1991). Voltage-gated Ca^2+^ channels have also been demonstrated in mouse oocytes (Murnane and De Felice 1993; Day et al. 1995) but because mouse oocytes show only negligible hyperpolarizations during fertilization (Igusa et al. 1983), it is unlikely that such channels mediate the Ca^2+^ influx.

In many cell types, the Ca^2+^ signal is biphasic: the release of Ca^2+^ from the intracellular stores is followed by Ca^2+^ influx across the plasma membrane. The mechanism is known as store-operated Ca^2+^ entry and it is a major signaling pathway in non-excitable cells (Putney 1986). In some cases, the extracellular Ca^2+^ entering the cell serves to keep cytoplasmic Ca^2+^ levels elevated and thus has a major role in the generation of the Ca^2+^ signal; in other cases, it helps to maintain the repetitive signal by refilling the intracellular stores (for a review, see Putney and Tomita 2012). In oocytes, it was also found that mobilizing the stored Ca^2+^ generated a Ca^2+^ influx across the plasma membrane. In mouse oocytes, depleting the intracellular stores with thapsigargin, an inhibitor of the SERCA pumps, activated a Ca^2+^ influx (Kline and Kline 1992). Ca^2+^ is known to slowly leak out of the endoplasmic reticulum via the ‘leak pathway’ and, because the blocked pumps are unable to reload Ca^2+^, the stores become depleted. The fact that store-depletion triggers a Ca^2+^ influx, without the activation of the phosphoinositide cascade, indicates that store-operated Ca^2+^ entry is functional in oocytes and may serve to refill the endoplasmic reticulum. Incubation in the presence of thapsigargin was later found to also stimulate Ca^2+^ entry in pig and human oocytes (Machaty et al. 2002; Martín-Romero et al. 2008), indicating that the mechanism might have a role in Ca^2+^ signaling.

Previous research suggested that the Ca^2+^ entry triggered by the filling status of the Ca^2+^ store is under the regulation of protein kinase C (PKC). 12-O-tetradecanoyl phorbol acetate (TPA) and phorbol-12-myristate-13-acetate (PMA) are phorbol esters that can very effectively stimulate PKC. When applied to mouse oocytes, they cause low amplitude Ca^2+^ oscillations and a variety of oocyte activation events downstream of the Ca^2+^ signal (Cuthbertson and Cobbold 1985; Endo et al. 1986; Colonna et al. 1989; Ducibella et al. 1991). PKC activation also promoted Ca^2+^ influx and repetitive Ca^2+^ oscillations (Yu et al. 2008), while constitutively active PKC constructs triggered a persistent elevation in cytosolic Ca^2+^ levels after the release of Ca^2+^ from the internal stores (Madgwick et al. 2005). In addition, 1-oleyl-2-acetyl-sn-glycerol (OAG), a synthetic analogue of endogenous diacylglycerol, the physiological activator of PKC, induces activation of mouse oocytes (Colonna et al. 1989). It has also been demonstrated that, in fertilized mouse oocytes, fluorescently labeled PKCs translocate repeatedly to the plasma membrane and the translocations occur in synchrony with the Ca^2+^ transients and the periodic increases in the rate of Ca^2+^ influx (Halet et al. 2004). On the other hand, inhibition of PKCs with bisindolylmaleimide I (BIM) blocks thapsigargin-induced Ca^2+^ influx and terminates the sperm-induced Ca^2+^ oscillations. It is possible that the Ca^2+^ entry channel in the plasma membrane or some accessory proteins are phosphorylated by PKC that in turn results in an increase in Ca^2+^ entry. These data imply that PKC is involved in the regulation of cytoplasmic Ca^2+^ levels in the oocyte, potentially by controlling a store-operated entry mechanism.

Store-operated Ca^2+^ entry in somatic cells is mediated by the interaction of two proteins, the stromal-interacting molecule (STIM) and Orai proteins. STIM1 and STIM2 are transmembrane proteins that reside in the endoplasmic reticulum (Liou et al. 2005; Roos et al. 2005). With an EF hand located inside the lumen of the store, they are able to sense its Ca^2+^ content. Upon Ca^2+^ mobilization, STIM1 forms small clusters (puncta), relocates to regions close to the plasma membrane and activates the Ca^2+^ entry channels. The channel is formed by Orai proteins (Orai1, Orai2 and Orai3). Orai1, the most potent isoform, resides in the oolemma and, once stimulated by STIM1, it allows Ca^2+^ in the extracellular medium to flow into the cell (Feske et al. 2006; Vig et al. 2006; Zhang et al. 2006). Both STIM1 and Orai1 have been identified in pig, mouse and frog oocytes (Koh et al. 2007; Gómez-Fernández et al. 2009, 2012; Yu et al. 2009; Wang et al. 2012). In the frog, where the fertilizing sperm triggers a single Ca^2+^ transient to activate the oocyte, store-operated Ca^2+^ entry is markedly down-regulated during maturation (Yu et al. 2009). In pig oocytes, the situation seems to be different. Down-regulation of STIM1 using siRNAs leads to a complete elimination of the Ca^2+^ oscillations associated with fertilization (Lee et al. 2012). Similarly, Orai1 knockdown inhibits store-operated Ca^2+^ entry and abolishes the sperm-induced Ca^2+^ transients (Wang et al. 2012), while specific inhibitors of store-operated Ca^2+^ entry were also effective in disrupting the repetitive Ca^2+^ signal at fertilization (Wang et al. 2015). This indicates that the Ca^2+^ influx that sustains the regenerative Ca^2+^ signal at fertilization is operated by the filling status of the stores and is mediated by STIM1 and Orai1 proteins.

Interestingly, inhibition of store-operated Ca^2+^ entry in mouse oocytes using pharmacological agents, or by preventing STIM1-Orai1 interaction through the expression of specific protein fragments, does not prevent the sperm-induced Ca^2+^ oscillations (Miao et al. 2012; Takahashi et al. 2013). This implies that mouse oocytes apply a Ca^2+^ entry mechanism other than that controlled by the intracellular store to maintain Ca^2+^ oscillations at fertilization. As mentioned above, the Ca^2+^ influx in the mouse seems to be under the control of PKC. A candidate channel to provide Ca^2+^ influx at fertilization is the transient receptor potential (TRP) channel. The TRP protein serves as a Ca^2+^ channel in a number of cell types and is expressed in various oocytes (Petersen et al. 1995; Machaty et al. 2002). Certain TRP isoforms are known to be regulated by PKC (Hardie 2007), which would account for the stimulatory effect of PKC on the sperm-induced Ca^2+^ signal. However, recent research has indicated that the TRP channel is not required for normal fertilization (Carvacho et al. 2013). Stimulation of TRPV3 channels leads to Ca^2+^ entry and subsequent oocyte activation but oocytes collected from transgenic mice that lack TRPV3 channels are able to generate the repetitive Ca^2+^ spikes characteristic of normal fertilization. This shows that TRPV3 is not essential to sustain the regenerative Ca^2+^ signal and thus the identity of the Ca^2+^ entry mechanism that operates in mouse oocytes at fertilization is still unclear.

## Future prospects

We have come a long way in the understanding of oocyte activation since Jacques Loeb’s “incredible discovery” and our knowledge regarding the signaling event that marks the formation of a new embryo has increased tremendously. We know that the fertilizing sperm triggers development by inducing an elevation in the oocyte’s cytosolic Ca^2+^ concentration. The source of Ca^2+^ is intracellular and, in mammals, the release is mediated by the phosphoinositide signaling system of the oocyte. It is also well accepted that the sperm stimulates this signaling cascade by introducing a soluble factor into the ooplasm after gamete fusion. Several lines of evidence support the idea that this key factor is phospholipase Cζ, a sperm-specific enzyme that, after gamete fusion, cleaves PIP_2_ and thus generates IP_3_ to mobilize stored Ca^2+^. PLCζ is fairly well characterized but we do not know how it finds PIP_2_ that resides in cytoplasmic vesicles, why it ignores PIP_2_ in the plasma membrane and whether or not its effect is mediated by a specific protein in the oocyte (Swann and Lai 2013). It is also unclear if it is the only sperm-derived oocyte-activating factor or if it acts in concert with other molecules such as PAWP, the latest addition to the sperm factor candidate list. Finally, the nature of the Ca^2+^ influx mechanism that is responsible to sustain the low-frequency Ca^2+^ oscillations also needs further clarification.

A better understanding of the signaling mechanism that operates at fertilization offers major benefits. Proper activation of the oocyte’s developmental program is critical not only during fertilization under normal physiological conditions but also for the success of a number of assisted reproductive technologies. Genetically modified animals have vast potentials and one powerful technology to generate such animals is somatic cell nuclear transfer. Artificial oocyte activation is an integral part of the technology; however, our inability to properly activate the reconstructed oocyte is one of the reasons for the extreme inefficiency of nuclear transfer procedures (Prather 1996). Because we do not completely know the underlying mechanism that mediates Ca^2+^ signaling in fertilized oocytes, we are not able to artificially induce the repetitive signals. Parthenogenetic activation methods generate Ca^2+^ changes that do not faithfully recapitulate those occurring after fertilization and this results in poor embryo development. Increasing our knowledge of how the sperm triggers the oscillatory Ca^2+^ signals will enhance our ability to more precisely control the process of signaling. Also, infertility in humans is a condition affecting more than 70 million (roughly 1 in 7) couples worldwide (Ledger 2008; Ombelet et al. 2008). Although a number of assisted reproductive technologies are available to alleviate the problem, conditions such as severe male factor infertility remain a formidable challenge. Intracytoplasmic sperm injection is a procedure that delivers the sperm directly into the ooplasm and, because it is highly effective in improving fertility rates, its popularity is now on a par with in vitro fertilization (Palermo et al. 2009). However, even this powerful technique can ensure only a clinical pregnancy rate of up to 45 % and the primary reason for the unsuccessful cycles is a failure in oocyte activation. Clinical data indicate that the activation deficiencies are associated with reduced levels of PLCζ (Yoon et al. 2008; Heytens et al. 2009) or PAWP (Aarabi et al. 2014b). PLCζ deficiency has been successfully counteracted with co-injection with mouse PLCζ mRNA (Yoon et al. 2008), while PAWP levels are also believed to have a predictive value in sperm fertilizing ability and, if confirmed to be an oocyte-activating factor, may also have application in the treatment of infertility (Aarabi et al. 2014b). Thus, the injection of a purified, active recombinant protein into the oocyte may have high therapeutic importance. The use of such a protein will also make it possible to assess how the extracellular medium shapes Ca^2+^ oscillations, as media composition reportedly affects Ca^2+^ signaling and subsequent embryo development (Dumollard et al. 2006; Banrezes et al. 2011). Characterizing the sperm factor that initiates the Ca^2+^ signal, along with the entire mechanism that is set in motion by the factor, will ultimately lead to the development of methods to effectively activate oocytes when spermatozoa are unable to do so.
